# Protocol to study secretome interactions using extracellular proximity labeling

**DOI:** 10.1016/j.xpro.2024.103509

**Published:** 2024-12-12

**Authors:** Joshua A. Rich, Sadeechya Gurung, Sasha Coates-Park, Yueqin Liu, Anshika Govil, William G. Stetler-Stevenson, David Peeney

**Affiliations:** 1 Laboratory of Pathology, Center for Cancer Research, National Cancer Institute, Bethesda, MD 20892, USA

**Keywords:** Biotechnology and bioengineering, Cell Biology, Mass Spectrometry, Proteomics

## Abstract

Biotin ligase-based proximity ligation is a widely used, highly effective technique for the study of *in vivo* protein-protein interactions. However, there are few reports and little consensus on the most effective methods for studying the proximal interactomes of secreted factors. Here, we present a protocol for studying extracellular proximal interactomes using an adaptation of TurboID/BioID2-based proximity ligation. We describe steps for cell preparation, sample collection, and initial processing. We then detail procedures for biotinylated protein enrichment, on-bead digestion, and post-pull-down processing.

For complete details on the use and execution of this protocol, please refer to Peeney et al.^1^

## Highlights

• Guidance on the production of secreted biotin ligase fusion proteins
• Steps for initiating extracellular proximity labeling experiments
• Instructions for processing unlabeled and isobaric labeled samples
• Best practices for data processing and interpretation

## Abstract (editor highlights)

Publisher’s note: Undertaking any experimental protocol requires adherence to local institutional guidelines for laboratory safety and ethics.

## Graphical abstract

**Figure:**
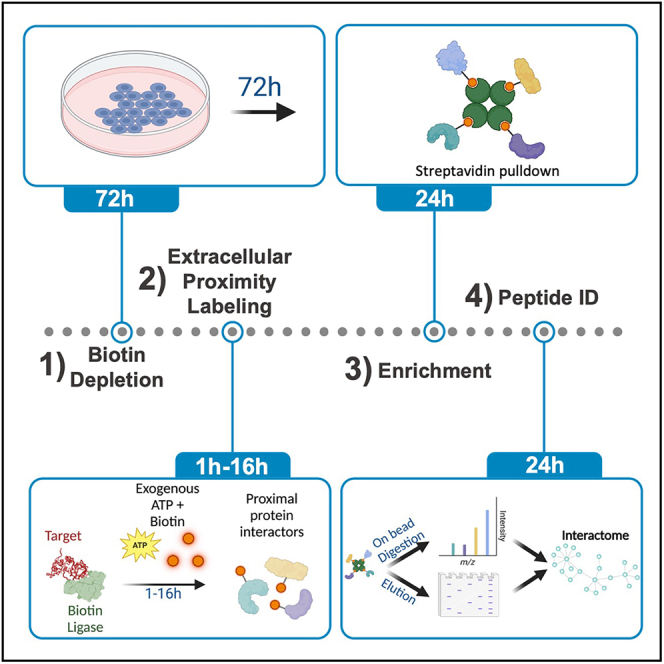

## Before you begin

The following protocol describes the step-by-step procedure for using proximity labeling *in vitro* to explore the proximal interactome of secreted factors. We utilized this protocol to study the TIMP2 interactome in HT1080 and HS-5 cells in both 2D and 3D cultures, and we have expanded this protocol to numerous other cell lines and targets.^1^

All biological processes are propagated through molecular interactions that overwhelmingly occur between components of the proteome. In humans, there are at least 18,000 individual protein identities, a number that soars when considering individual proteoforms.^2^ Interactome studies provide insights into the functions and subcellular localization of target proteins, the findings of which have proven invaluable across basic and translational biomedical research. The relatively recent proliferation of interactome studies using proximity labeling (PL) has provided researchers with a powerful tool for studying proximal protein-protein interactors (prPPIs) in living systems. These methods rely on the presence or expression of a fusion consisting of the target protein linked to a biotin ligase or an engineered ascorbate peroxidase, the results of which produce complimentary findings to those of classical methods such as affinity purification/mass spectrometry (AP/MS) and yeast 2-hybrid (Y2H).^3^ PL has several key advantages over traditional interactome study methods, including the ability to capture weak/transient interactions, flexibility with reaction kinetics depending on the chosen PL enzyme, and a reliance on the potent biotin-streptavidin interaction. The biotin-streptavidin interaction is a double-edged sword with regards to its role in the power of PL. On one hand, the strong interaction allows for extensive washing to remove contaminants. On the other hand, dissociation of the interaction to elute interactors requires harsh conditions that can be incomplete and detrimental to downstream analysis.

The matrisome is the extracellular matrix (ECM) proteome, an ensemble of macromolecules that maintain tissue architecture and govern tissue function. Extensive effort and resources have been apportioned to explore the matrisome in normal and diseased tissues, which are hampered by the distinct biochemical properties of the ECM proteome that include large, cross-linked, and/or poorly soluble proteins.^4^ PL is overwhelmingly performed on intracellular targets, a favorable reaction that relies on readily available ATP to catalyze biotin labeling. Herein we describe a complete method for the interrogation of extracellular PPIs using BioID2 and TurboID, an adaption that we term extracellular proximity labeling (ePL).

### Fusion gene cloning and cell line generation

**Timing: 2+ weeks**

**CRITICAL:** N- and C-terminal tagged constructs should be generated (Figure 1A), as this can have a profound effect on the identified proximal interactome. Furthermore, expression of the fusion proteins and control (biotin ligase alone) should be comparable in target cell lines. High expression of the fusion proteins is desirable, as secretion into the extracellular compartment produces a substantial dilution factor.

***Alternatives:*** Constructs can be designed with or without the 13X GS-linker, which increases the labeling range.^5^ Without a flexible linker biotin ligases have a labeling range of approximately 10 nm and above (depending on overall protein structure).^5^ We used a retroviral expression system (with a pBABE backbone), though other gene transfer systems are also suitable. The choice between biotin ligases should be empirical to the aims (fast labeling kinetics versus slow labeling kinetics).^6^ Target stability, recycling, and degradation should be considered. We noted that MMP14, a membrane receptor for TIMP2, could only be detected as a prPPI with TurboID.^1^

1. Clone retroviral expression vectors encoding fusion constructs for control (secretory signal peptide + biotin ligase), N-terminal target fusion (secretory signal peptide + biotin ligase + GS-linker + target [without endogenous signal peptide]), and the C-terminal target fusion (target + GS-linker + biotin ligase).

**Figure 1:**
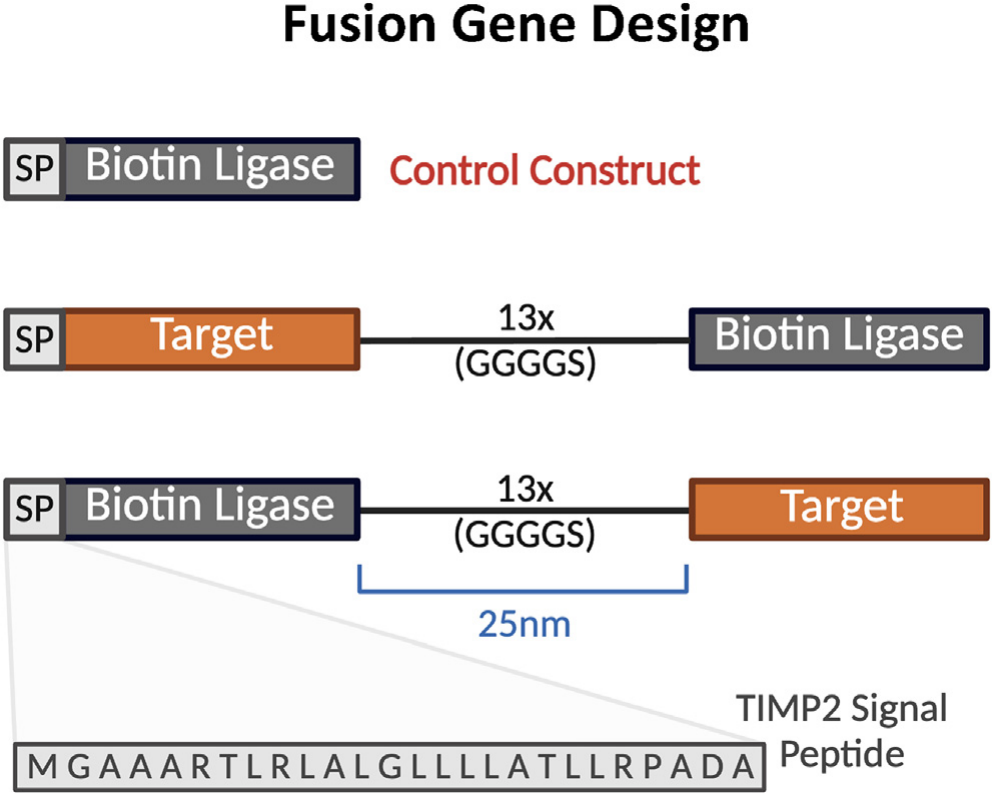
Fusion gene orientation with a 25 nm 13x GS (GGGGS) linker
2. Generate stable fusion gene expressing cell lines.
  a. Assess for expression levels, secretion, and (if possible) functional retention.

### Generate biotin depleted full media and low-serum biotin depleted media

**Timing: 1 day**

***Note:*** Different batches of serum may have differing levels of free biotin. It is vital that the end-user test the biotin depletion protocol works for their serum formulation.

***Alternatives:*** Researchers may use dialyzed serum; however this may have pronounced effects on cell growth and viability.

3. Add 1.25 mL high-capacity streptavidin agarose resin to a 50 mL tube. Make the volume up to 50 mL with Dulbecco’s phosphate buffered saline (DPBS) and centrifuge at 1000 x G for 2 min.
4. Carefully remove most of the supernatant and repeat this wash step for a total of 4 washes.
5. Resuspend the washed resin in 5 mL full media. Transfer the resin and approximately 550 mL of FM to a 1 L roller bottle.
  a. Roll the bottle for 4–20 h at 4°C.
6. Remove the resin by filtration through a 0.2 μm polyethersulfone membrane. This is BDFM.
7. To generate LSBDM, combined 1-part BDFM with 9 parts Advanced DMEM, 1% penicillin-streptomycin, 1% GlutaMAX. Medias are stable for 1 month at 4°C.

### Optimize proximity labeling conditions

**Timing: 3+ weeks**

**Figure 2:**
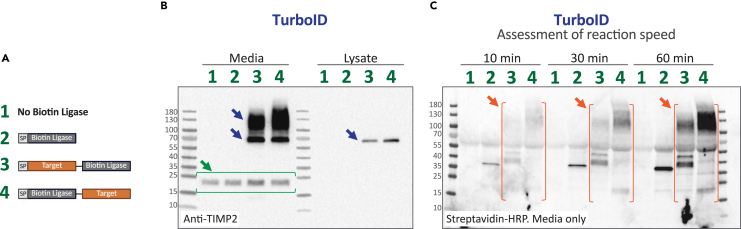
Expected expression levels and biotinylation patterns of an ePL experiment (A) Key illustrating constructs utilized. (B) Expression levels of the TIMP2-TurboID fusion proteins (blue arrows) versus endogenous TIMP2 (green arrows). Recombinant TIMP2 proteins often dimerize when at high abundance (higher molecular weight bands). (C) Expected biotinylation patterns in conditioned media collected from a TurboID experiment at 10, 30, and 60 min. Unique biotinylated protein signatures are highlighted in orange.

***Note:*** Although we describe the optimized conditions, material batch effects and supplier differences may require additional rounds of optimization. For optimization, it is logical to utilize a single target fusion and the control. An example of the expected relative expression of fusion proteins and patterns of biotinylation in ePL assays are illustrated in Figure 2.

8. Using a known (ideally strong) interactor, optimize the conditions for proximity labeling.
  a. Perform small-scale ePL reactions with 100% confluent cells (to maximize yield) followed by pulldowns with streptavidin magnetic beads.
  b. Optimize biotin concentration. We determined 1 μM to be the optimal for labeling, other groups use 50 μM. It is suggested to try a range between 0.1 – 100 μM.
  c. Optimize ATP concentration. This is biotin ligase-dependent. We utilized 0.1 mM ATP for TurboID, and 2 mM for BioID2. It is suggested to try a range between 10 μM and 4 mM.
  d. Elute biotinylated proteins by boiling for 5 min using 4X Laemmli buffer + 20mM dithiothreitol (DTT) + 2mM biotin. Analyze samples through SDS-PAGE followed by immunoblotting for the strong interactor, and blotting with streptavidin-horse radish peroxidase (HRP) to assess biotinylation patterns. The control construct samples should not produce biotinylation of known target interactors.

### Produce trypsin-resistant streptavidin

**Timing: 1 day**

**CRITICAL:** Sodium cyanoborohydride is a volatile poison and should be handled with extreme care. This chemical is extremely hygroscopic, making handling difficult under ambient conditions. It is recommended that single-use 100 mg aliquots be prepared under inert conditions. Following reaction completion, remaining reactant and buffers must be safely disposed of based upon the institution’s guidelines. This step is based on the protocol by Rafiee et al.^7^ It is highly recommended to use magnetic beads, versus polysaccharide beads due to the superior ease of washing.

***Alternatives:*** Instead of trypsin-resistant streptavidin, users can elute by boiling for 5 min in an SDS buffer (2% SDS, 50mM Tris-HCl, 10mM DTT, 2mM biotin) and perform a cleanup step before tryptic digestion such as SP3 cleanup.^8^ We found SP3 cleanup to be as effective as trypsin-resistant streptavidin in these assays.

9. Prepare trypsin-resistant streptavidin beads.
  a. Add 0.66 mL streptavidin beads into 3x 2mL centrifuge tubes.
  b. Place on magnet, wait 5 min, discard supernatant.
  c. Wash beads with 1.32 mL PBS-T.
  d. Place on magnet, wait 5 min, discard supernatant.
  e. Resuspend beads in 1.87 mL reagent CHD.
  f. Rotate at 19–23°C for 4hr.
  g. Place on magnet, wait 5 min, discard supernatant.
  h. Wash beads with 1.32 mL PBS-T.
  i. Place on magnet, wait 5 min, discard supernatant.
  j. Resuspend beads in 0.93 mL reagent A.
    ***Note:*** TOXIC - perform in chemical fume hood.
  k. Add 0.93 mL reagent B.
    ***Note:*** TOXIC - perform in chemical fume hood.
  l. Rotate at RT for 2 h.
  m. Place on magnet, wait 5 min, discard supernatant safely.
  n. Wash beads with 1.32 mL 0.1 M Tris-HCl, pH 7.5.
  o. Place on magnet, wait 5 min, discard supernatant safely.
  p. Wash beads with 1.32 mL PBS-T.
  q. Place on magnet, wait 5 min, discard supernatant safely.
  r. Resuspend beads in 0.66 mL PBS-T.
  s. Store beads at 4°C. They are stable for at least 12 months if kept sterile.

## Key resources table

**Table:** 

REAGENT or RESOURCE	SOURCE	IDENTIFIER
**Antibodies**		

*A. aeolicus* BPL/BioID2 antibody (1:2,000 dilution)	Novus Biologicals	NBP2-59941
V5 tag (1:2,000 dilution)	Cell Signaling Technology	13202
TIMP2 (1:2,000 dilution)	Cell Signaling Technology	5738

**Chemicals, peptides, and recombinant proteins**		

10% SDS (ultrapure)	Thermo Fisher Scientific	J77504-AP
5M sodium chloride	Quality Biological	351-036-101
Adenosine 5′-diphosphate disodium salt hydrate (ATP)	MilliporeSigma	A6419-5G
Advanced DMEM	Gibco	12491-015
Anhydrous acetonitrile	Thermo Fisher Scientific	042311-AK
Biotin	MilliporeSigma	B4501
Cyclohexanedione	MilliporeSigma	C101605
DL-dithiothreitol (DTT)	MilliporeSigma	D5545
Dulbecco’s modified Eagle’s medium (DMEM)	Quality Biological	112-013-101
Dulbecco’s phosphate-buffered saline with magnesium and calcium (DPBS)	Gibco	14040-133
EDTA, 0.5 M stock (UltraPure)	Invitrogen	15575-038
Fetal bovine serum (FBS)	Gemini Bio-Products	100-106
Formic acid, 99%	Thermo Fisher Scientific	270480010
GlutaMAX supplement	Gibco	35050-061
HEPES buffer, 1 M	Gibco	15630-080
High capacity streptavidin agarose resin	Thermo Fisher Scientific	20361
Hydroxylamine (50%)	Thermo Fisher Scientific	90115
Iodoacetamide	MilliporeSigma	I1149-25G
Laemmli sample buffer (4x)	Bio-Rad	1610747
LC/MS-grade water	Thermo Fisher Scientific	T51140-K2
Lipofectamine LTX reagent	Invitrogen	94756
Lithium chloride	MilliporeSigma	310468
Opti-MEM	Gibco	31985-070
Paraformaldehyde ampoules (16%)	Electron Microscopy Sciences	15710
Penicillin-streptomycin, 10,000 U/mL	Gibco	151-10-122
PLUS reagent	Invitrogen	10964-021
Polybrene	MilliporeSigma	TR-1003-G
Protease inhibitor cocktail (PIC)	MilliporeSigma	P8340-5ML
Sera-Mag SpeedBeads streptavidin-blocked magnetic particles	Cytiva	21152104010150
Sodium cyanoborohydride	MilliporeSigma	156159
Sodium deoxycholate	MilliporeSigma	30970-100G
Sodium hydroxide	MilliporeSigma	S8045
Streptavidin-HRP	Cell Signaling Technology	3999
Surfact-Amps NP-40	Thermo Fisher Scientific	28324
Surfact-Amps Triton X-100	Thermo Fisher Scientific	28314
TMT10plex	Thermo Fisher Scientific	90110
Triethylammonium bicarbonate (TEAB) buffer, 1.0 M	MilliporeSigma	T7408-100ML
Trifluoroacetic acid (TFA)	MilliporeSigma	302031
Tris buffer, 1 M, pH 8.0	K·D Medical	RGF-3360
Tris-HCl buffer, 1 M, pH 7.5	Corning	46-030-CM
Trypsin (0.25%)-EDTA	Gibco	25200-056
Trypsin/Lys-C mix, mass spec grade	Promega	V5071
Tween 20, ultrapure	Thermo Fisher Scientific	J20605-AP

**Critical commercial assays**		

QIAprep spin miniprep kit	QIAGEN	27104
Protein quantification assay	MACHEREY-NAGEL	740967.50

**Experimental models: Cell lines**		

HS-5 bone marrow stromal cell line	ATCC	CRL-3611
HT1080 fibrosarcoma cell line	ATCC	CCL-121
Retrovirus-producing cell line: HEK 293T/17	ATCC	CRL-11268

**Recombinant DNA**		

pVSV-G expression plasmid	Addgene	138479
pUMVC packaging plasmid	Addgene	8449
Target:TurboID pBABE expression plasmid	This study	Parent source: Addgene #80900, #80899, #107173
TurboID:target pBABE expression plasmid	This study	Parent source: Addgene #80900, #80899, #107173
TurboID control pBABE expression plasmid	This study	Parent source: Addgene #80900, #80899, #107173
Target:BioID2 pBABE expression plasmid	This study	Parent source: Addgene #80900 and #80899
BioID2:target pBABE expression plasmid	This study	Parent source: Addgene #80900 and #92308
BioID2 control pBABE expression plasmid	This study	Parent source: Addgene #80900 and #92308

**Software and algorithms**		

Proteome Discoverer. Alternative: MaxQuant is a free alternative	Thermo Fisher Scientific	–

**Other**		

Amicon Ultra 15 mL centrifugal filters, 10K MW cutoff	MilliporeSigma	UFC901096
Amicon Ultra 4 mL centrifugal filters, 10K MW cutoff	MilliporeSigma	UFC801096
Benchtop centrifuge (for 15 mL/50 mL tubes)		
C18 spin tips, 100 μL	Thermo Fisher Scientific	87784
Cell lifters	Corning	3008
ChemiDoc Imaging System. Alternative: many alternatives are available.	ChemiDoc MP	–
DynaMag-2 magnet	Thermo Fisher Scientific	12321D
Microcentrifuge		
NanoDrop One UV-vis spectrophotometer. Alternative: many alternatives are available.	Thermo Fisher Scientific	ND-ONE-W
PD-10 columns	Cytiva	17085101
Polystyrene storage (roller) bottles	Corning	8396
Protein LoBind tubes, 1.5 mL	Eppendorf	022431081
Protein LoBind tubes, 2 mL	Eppendorf	022431102
Protein LoBind tubes, 5 mL	Eppendorf	0030122356
Sonicator cup horn	Qsonica	431C2
Vacuum filtration system, pore size 0.22 μm, polyethersulfone membrane	MilliporeSigma	S2GPU11RE
Water bath sonicator. Alternative: many alternatives are available.	Qsonica	Q500
Vacuum concentrator; SpeedVac or similar.	Thermo Fisher Scientific	–

## Materials and equipment

**Table:** PBS-T

Reagent	Final concentration
Phosphate buffered saline	–
Tween-20	0.1%

**Table:** PBS-T, pH 13

Reagent	Final concentration
Phosphate buffered saline	–
Tween-20	0.1%

***Note:*** Adjust pH with sodium hydroxide

**Table:** Reagent A

Reagent	Final concentration
Paraformaldehyde	4%
PBS-T	–

**CRITICAL:** Toxic. Perform in a fume hood. Generate 8 mL

**Table:** Reagent B

Reagent	Final concentration
Sodium cyanoborohydride	0.2 M

**CRITICAL:** Toxic. Perform in a fume hood. Generate 8 mL

**Table:** Reagent CHD

Reagent	Final concentration
Cyclohexanedione	76.5 mM
PBS-T, pH 13	–

***Note:*** Generate 14 mL

**Table:** Biotin stock

Reagent	Final concentration
Biotin	125 mM
HEPES	1M

***Note:*** Incubate at 55°C for 15 min and vortex. Sterile filter and store aseptically for up to 12 months at 4°C.

**Table:** Full media

Reagent	Final concentration
DMEM	1X (500 mL)
FBS	8.9% (50 mL)
GlutaMAX	1% (5.5 mL)
Penicillin-Streptomycin	1% (5.5 mL)

***Note:*** Store aseptically at 4°C for up to 1 month.

**Table:** ATP stock

Reagent	Final concentration
ATP	100 mM
DMEM	–

***Note:*** Make ∼1 mL fresh.

**Table:** Lysis buffer

Reagent	Final concentration
HEPES	50 mM
NaCl	50 mM
SDS	2%
Triton X-100	1%
NP-40	1%
Sodium deoxycholate	1%
LC/MS-grade water	–

***Note:*** Store aseptically at 4°C for up to 12 months. Before use, add 1% protease inhibitor cocktail and 10 mM DTT.

**Table:** Wash 1

Reagent	Final concentration
SDS	2%
LC/MS-grade water	–

***Note:*** Make fresh.

**Table:** Wash 2

Reagent	Final concentration
Sodium deoxycholate	0.1%
Triton X-100	1%
NaCl	500 mM
EDTA	1 mM
HEPES	50 mM
LC/MS-grade water	–

***Note:*** Store aseptically at 4°C for up to 12 months.

**Table:** Wash 3

Reagent	Final concentration
LiCl	250 mM
NP-40	0.5%
Sodium deoxycholate	0.5%
EDTA	1 mM
Tris pH 8	10 mM
LC/MS-grade water	–

***Note:*** Store aseptically at 4°C for up to 12 months.

**Table:** Wash 4

Reagent	Final concentration
HEPES	20 mM
NaCl	150 mM
LC/MS-grade water	–

***Note:*** Store aseptically at 4°C for up to 12 months.

**Table:** TEAB buffer

Reagent	Final concentration
TEAB	100 mM
LC/MS-grade water	–

***Note:*** Make fresh.

## Step-by-step method details

**Figure 3:**
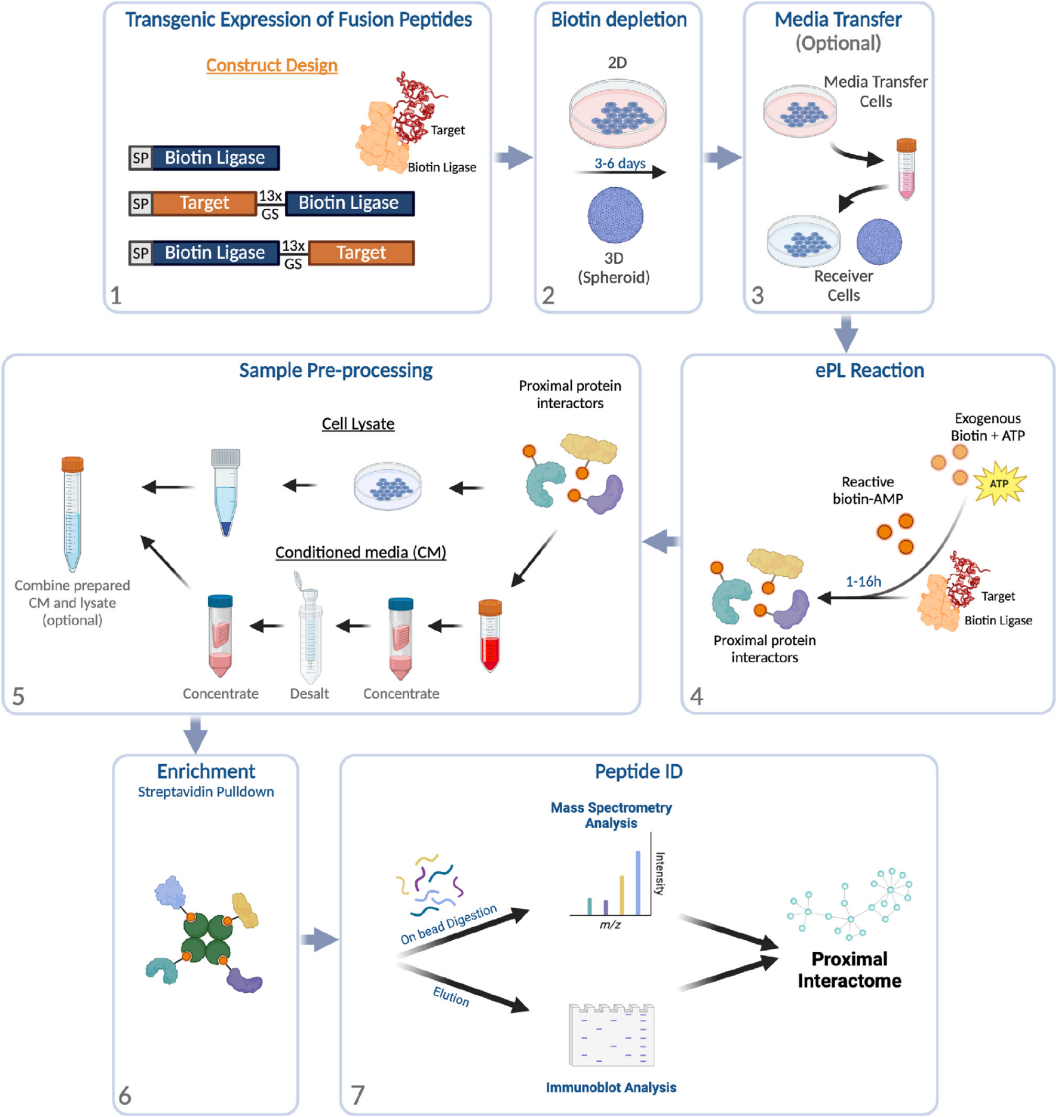
Extracellular proximity labeling summarized workflow

The workflow in Figure 3 summarizes the steps involved in ePL performed with HT1080 fibrosarcoma cells and HS-5 bone marrow stromal cells. It is recommended to utilize a ‘conditioned media transfer’ method, whereby 24h-conditioned media from fusion gene-expressing cells is transferred on top of wild type (WT) cells at the initiation step. This significantly limits intracellular off-target labeling in this high-expression system.

### Cell preparation

**Timing: 5 days**

1. Before starting, be sure you have a confluent 150 mm tissue culture dish of each cell line.
  a. Wash the cells 3x with DPBS before detachment with 1000 μL trypsin/EDTA.
  b. Resuspend the cells in 10 mL BDFM for counting.

***Note:*** Cell numbers need to be optimized for new cell lines. We aimed for 100% confluent cells at the start of the proximity labeling reaction to maximize yield.

2. Seed 1.5 x 10^6^ of each transgene-expressing cell line into a single 150 mm culture plate. These are Media Transfer Cells (MTCs).
3. Next, seed 1.5 x 10^6^ WT cells in 5 × 150 mm culture plates. These are the 4 x Receiver Cells (RCs) and 1 x WT MTC. All cells should be seeded in 25 mL BDFM.
  Single experiment required cells:
  a. WT control MTC
  b. Biotin ligase control MTC
  c. Target-Biotin ligase MTC
  d. Biotin ligase-Target MTC
  e. WT RC x 4
    ***Note:*** WT control MTC and 1 x WT RC can be excluded to reduce sample burden at the expense of reduced controls. WT control MTC should be excluded if using tandem mass tag (TMT)-labeling, since lower biotinylated protein concentration in these samples will dramatically skew the results after normalization prior to labeling.
4. Incubate cells for 3 days at 5% CO_2_ and 37°C (Figure 3, step 2).
5. Replace 10mL of media on the RCs with fresh BDFM to keep the RCs in healthy condition.
6. Replace the media on the MTCs with 23 mL LSBDM, then incubate the MTCs for 24 h at 5% CO_2_ and 37°C to complete serum starvation.

***Note:*** This step is important to minimize serum protein levels while retaining cell viability.

***Note:*** Keep in mind the reaction times of different biotin ligases when completing this step since the ePL reaction will be initiated 24 h later. For example, for the 1-h reaction of TurboID, a morning media change is advisable. On the contrary, a late afternoon media change is advisable for the 16-h BioID2 reaction.

***Note:*** Different cell types will respond in different ways to serum reduction. Users should test target cell line responses to these conditions.

7. After 24 h serum starvation of the MTCs, aspirate all media from the RCs and immediately transfer the conditioned media (CM) from the MTCs (approximately 22 mL after evaporative loss) to the RCs (Figure 3, step 3).
8. Initiate the ePL experiment by adding ATP solution and biotin solution to the appropriate concentrations (Figure 3, step 4).

***Note:*** The working ATP concentration for TurboID is 0.1 mM, while for BioID2 it is 2 mM. For both TurboID and BioID2, the working biotin concentration is 1 μM.

### Sample collection and initial processing

**Timing: 0.5–1 day**

***Note:*** This section includes two parts, the collection of CM and the collection of cell lysate. Depending on experiment size and user comfort, it is advisable for two separate researchers to perform these parts in parallel to minimize the adverse effects of delaying either part.

### Conditioned media collection

9. At end of the ePL reaction, transfer as much CM from the cells as possible to 50 mL conical tubes and supplement with 0.1% protease inhibitor cocktail.

**CRITICAL:** Immediately cool media on ice and keep it cool to inhibit excess biotin labeling.

10. Centrifuge at 2,000 x g for 5 min at 4°C to pellet any floating cells/detritus.
11. Gently pour the centrifuged CM into new 50 mL conical tubes, leaving behind the pellet.
12. Concentrate the CM to ∼1 mL using Amicon 15 concentrators (10 kDa) at 4,300 x G, 4°C, for approximately 30 min.

***Note:*** This will need to be done in two spins using the same concentrator with flow-through discarded between the two spins, since the maximum concentrator volume is 15 mL.

13. To remove free biotin, desalt the concentrated media by gravity flow using PD-10 columns per the manufacturer’s instructions. The buffer should be exchanged to DPBS, and the returned sample will be in a 3.5 mL volume. A brief gravity protocol is described below.
  ***Note:*** Do not allow PD-10 columns to dry.
  a. Clean PD-10 columns (one per sample) with 25 mL DPBS.
  b. Measure the volume of the concentrated samples, then add to the PD-10 columns. Allow sample to flow into the column completely.
  c. To each column, add DPBS to make each loaded volume up to 2.5 mL. Allow this to fully enter the column.
  d. Move PD-10 columns to the elution collection tubes, then load 3.5 mL of DPBS. The flow through is the eluent.
14. Concentrate samples to ∼1000 μL using Amicon 4 concentrators (10 kDa) at 4,300 x G, 4°C, for approximately 12 min.
15. Transfer the concentrated sample to a 5 mL tube and keep on ice (see stopping point below).

### Cell lysate collection

16. On ice, wash the cells 3x with 12 mL ice cold DPBS (with Mg^2+^ and Ca^2+^).
  a. At the final wash, remove excess DPBS by tilting the culture plate for 10 s before aspiration.

**CRITICAL:** 3x thorough washes are important to remove residual biotin.

17. Add 1250 μL 19–23°C lysis buffer to the cells. Harvest by scraping and add the resulting sample to a 2 mL low-binding tube.

***Note:*** Sample will be viscous. Using cold lysis buffer will make the viscosity worse.

18. Mix the samples by vortexing (10 × 2 s at high power).
19. Heat samples in a heat block set to 95°C for 10 min while shaking at 800 RPM.
20. Incubate on ice for 3 min to cool the samples.
21. Sonicate samples with 24 × 5 s bursts at 80% power with RT distilled water.
22. Pellet any insoluble debris at 10,000 x G for 10 min at 15°C.

***Note:*** The lysis buffer is very effective at solubilizing cell components, so you will likely have little/no pelleted debris.

23. Transfer the supernatant to the same 5 mL tubes for the corresponding samples in step 7.

*Alternative:* CM and lysate samples can be analyzed independently. If you choose to do this, do not pool the samples.

***Note:*** Post-pulldown sample concentration can be very low, which can be detrimental to proteomic analysis. Pooling CM and lysate helps to increase input for LC-MS/MS analysis.

24. Mix the samples with a light vortex. Over-vortexing can create many bubbles which is undesirable for freezing.
  a. Snap-freeze the tubes in liquid nitrogen.

***Note:*** Snap-freezing helps to significantly reduce the viscosity of the samples, which is important for future steps.

### Stopping point

Sample processing can continue immediately or be performed another day.

### Biotinylated protein enrichment (pull-down) and on-bead digestion

**Timing: 1 day**

25. Thaw samples in a 37°C water bath.
26. Add 60 μL protease-resistant streptavidin resin to each tube. Mix by inversion and vortexing. Incubate at 4°C for 2 h on a rotator (Figure 3, step 6).
27. After incubation with the resin is complete, spin the tubes at 500 x G for 20s to remove sample from the lid.
28. Divide each sample into 2 x pre-labeled 2 mL low-binding tubes. Place the tubes on the magnetic rack and incubate for 5 min. Discard the supernatant.

**CRITICAL:** A 5 min incubation is required since the sample is viscous.

29. Remove the tubes from the magnetic rack. Using 1000 μL wash 1, resuspend the resin from one of the tubes and move the resin to the other tube for the corresponding sample, mixing up and down to resuspend the remaining resin.
  a. Discard the empty tube. Repeat for all samples, then place the tubes back on the magnetic rack, incubate for 1 min, and remove the supernatant.

**CRITICAL:** For this and the remaining wash steps, it is vital that the resin is fully resuspended with pipetting or vortexing. Failure to do so will increase background signal.

30. Take samples off the magnetic rack, wash the resin with another 1000 μL wash.
  a. Incubate on the magnetic rack for 1 min, then remove the supernatant.
31. Perform a single wash using 1000 μL wash 2.
  a. Incubate on the magnetic rack for 1 min, then remove the supernatant.
32. Perform a single wash using 1000 μL wash 3.
  a. Incubate on the magnetic rack for 1 min, then remove the supernatant.
33. Resuspend the resin in 1000 μL wash 4.
34. Transfer 50uL of washed resin from each sample to a fresh 1.5 mL lo-bind tube for in-house elution and testing. Snap-freeze the 50 μL sample or immediately process:
  a. Add the in-house testing tubes to the magnetic rack. Incubate for 1 min.
  b. Remove supernatant and resuspend in 50 μL 4X Laemmli sample buffer supplemented with 2 mM biotin and 20 mM DTT. Heat at 95°C for 5 min.
  c. Centrifuge the samples briefly to bring the beads to the bottom of the tube, then add the tubes to the magnetic rack. Remove the supernatant for storage at −20 or immediate analysis by SDS-PAGE.
35. Place the remaining 950 μL samples on the magnetic rack and remove the supernatant.
36. Perform another wash using 1000 μL wash 4.
  a. Incubate on the magnetic rack for 1 min, then remove the supernatant.
37. Wash the beads with 500 μL 100 mM Triethylammonium bicarbonate (TEAB).
  a. Before placing the tubes back in the magnetic rack and removing the supernatant, transfer the resuspended beads to fresh 1.5mL protein low-binding tubes.
38. Reduction step: Off the magnetic rack, resuspend the beads in 50 μL 100 mM TEAB supplemented with 5 mM DTT. Incubate the samples at 56°C for 25 min.
39. Alkylation: Make up fresh 0.5 M iodoacetamide solution in LC/MS-grade water. Add this solution to each sample to a final iodoacetamide concentration of 15 mM (1.55 μL).
  a. Briefly mix the samples by lightly pipetting up and down, then incubate the samples at 19–23°C for 30 min in the dark.
40. Place the samples on the magnetic rack, incubate for 1 min, and remove the supernatant.
41. Wash the beads 3x using 200 μL 100 mM TEAB for each wash.
42. Tryptic digestion: Assuming 2.5 μg protein, resuspend the beads in 30 μL 100 mM TEAB before adding 0.125 μg trypsin/LysC (0.5 μL of 0.25 μg/μL stock) to each sample.
  a. Gently mix the samples by pipetting up and down (Figure 3, step 7).

***Note:*** It is suggested that preliminary testing be performed to determine the expected amount of protein bound to magnetic beads in the user’s system. To do this, perform a preliminary experiment to elute protein from the magnetic beads and perform a Protein Quantification Assay (Machery Nagel) to determine total protein bound to the beads. Adjust trypsin/LysC amount based on the total protein to maintain a 1:20–1:50 protease:protein ratio.

43. Incubate the samples for 16–18 h at 37°C with shaking at 500 RPM.
44. Spin the samples at 500 x G for 20s to remove any condensation from the lids, then return the samples to the magnetic rack.

### Post-pull-down processing

**Timing: 30 min–2 h**

This section includes two methods, one for processing label-free samples (step 45) and one for processing with TMT labeling (step 46). Before analysis by LC-MS/MS, samples from both methods should be dried using a vacuum centrifuge and resuspended in 16 μL 0.1% formic acid.

45. Label-free processing:
  a. Transfer the ∼30 μL supernatant (small losses are expected due to evaporation) into new 1.5 mL protein low-binding tubes.
  b. To maximize yield, wash the beads with 30 μL 100 mM TEAB and place on the magnetic rack.
  c. Combine the supernatant with the supernatant from the previous step. The tubes with the beads may now be discarded.
  d. To clean any residual contamination with magnetic beads, return the samples to the magnet and incubate for 1 min.
    i. Transfer the supernatant to new 1.5 mL protein low-binding tubes.
  e. Add 3 μL 10% (v/v) trifluoroacetic acid (TFA) to each sample for a total sample volume of about 63 μL. Gently mix the samples by pipetting up and down.
  f. Snap-freeze samples in liquid nitrogen. The samples are ready for mass spectrometric analysis.
46. TMT-labeled processing
  a. Transfer the ∼30 μL supernatant (small losses are expected due to evaporation) into new 1.5 mL protein low-binding tubes.
  b. To maximize yield, wash the beads with 15 μL 100 mM TEAB and place on the magnetic rack. Then combine the supernatant with the supernatant from the previous step.
    i. The tubes with the beads may now be discarded.
  c. Quantify peptides using 1.5 μL of each sample with a spectrophotometer using absorbance at 205 nm.
    i. Scopes Method; minimum concentration detectable = 5 ng/μL. Absorbance is proportional to concentration, so normalization can be performed directly from A205 values.
  d. Normalize samples to the lowest quantity of protein (within each 3-plex experiment). Use 100 mM TEAB for normalization.
    ***Optional:*** To control for sample input prior to labeling, precisely add 0.5 μL of 40 ng/μL digested E. coli Maltose Binding Protein (MBP) standard (or another peptide standard)
  e. Add 22 μL anhydrous acetonitrile (ACN) to each 0.8 mg TMT label and mix by pipetting. Incubate at 19°C–23°C in the dark for 5 min.
  f. Add 5 μL TMT label to each sample (equates to 3.64 μg/μL = 10.7 mM). Mix by pipetting and incubate at 19°C–23°C in the dark for 1h.
    ***Note:*** The remaining TMT labels may be stored as 5 μL aliquots at −80°C for 1 month. Be sure to note which TMT label is used for each sample.
  g. Add 1 μL 15% (v/v) hydroxylamine (0.3% final) to each tube. Mix by pipetting.
  h. Add 1.7 μL 10% (v/v) TFA to the samples to acidify, bringing the total sample volume to 52.7uL. Mix by pipetting.
  i. Combine all samples into a single tube.
  j. C18-based sample cleanup and concentration:
    ***Note:*** Use individual tubes for each wash step. Elution begins immediately following submersion into elution buffer. The elution buffer should be generated in the “elution tube” to prevent sample loss.
    i. Prepare the “elution tube” by dispensing 100 μL elution buffer (0.1% (v/v) formic acid, 95% (v/v) ACN in LC/MS-grade water) into a 1.5 mL protein low-binding tube.
    ii. Wet the pipette tip by aspirating 100 μL of 50% (v/v) ACN in LC/MS-grade water and then discarding the solvent. Repeat once.
    iii. Equilibrate the tip by aspirating 100 μL of 0.1% (v/v) TFA in LC/MS-grade water and discarding the solvent. Repeat once.
    iv. Aspirate 90 μL of sample into the C18 tip, which binds the peptides to the C18 resin. Slowly dispense the liquid into a clean tube, then aspirate and dispense 5 times. The final dispense should go into a clean waste tube.
    v. Repeat the above step until all of the sample has been used and the peptides are now immobilized in the C18 resin of the tip.
    vi. Rinse the tip by aspirating 100 μL of 0.1% TFA/5% ACN in LC/MS-grade water and discarding the solvent. Repeat once with fresh 100 μL of 0.1% TFA/5% ACN solution.
    vii. To elute the sample, slowly aspirate 100 μL elution buffer from the elution tube, then slowly dispense it back into the same tube for snap-freezing.

## Expected outcomes

Transgenes should be expressed and secreted in target cell lines at comparable levels. Loss of immunoreactivity is possible with fusion proteins, so it is suggested that multiple antibodies are utilized targeting different components of the fusion protein (target, biotin ligase, epitope tag). Abundance should be high since significant dilution occurs upon secretion to the extracellular space. Streptavidin-HRP blotting of cell lysates/CM and biotin-enriched samples should reveal clear, unique biotinylated protein signatures in biotin ligase-expressing cells. Known, strong interactors should be used for validation using pulldowns and immunoblotting prior to LC-MS/MS analysis.

## Quantification and statistical analysis

**Timing: 1 day**

***Note:*** The following is an example analysis procedure performed by Peeney et al. using a Thermo Scientific Orbitrap Exploris 240 Mass Spectrometer and a Thermo Dionex UltiMate 3000 RSLCnano System.^1^ Before analysis, samples should be dried using a vacuum concentrator and resuspended in 16 μL 0.1% formic acid, with 12 μL being analyzed by liquid chromatography and tandem mass spectrometry (LC-MS/MS). The LC-MS/MS analysis of tryptic peptides for each sample should be performed sequentially with a blank run between each two sample runs. Sample should be loaded onto a peptide trap cartridge at a flow rate of 5 μL/min. The trapped peptides are then eluted onto a reversed-phase Easy-Spray Column PepMap RSLC, C18, 2 μM, 100 A, 75 μm × 250 mm (Thermo Scientific) using a linear gradient of acetonitrile (3–36%) in 0.1% formic acid. The elution duration should be 110 min at a flow rate of 0.3 μL/min. Eluted peptides are then ionized and sprayed into the mass spectrometer, using a Nano Easy- Spray Ion Source (Thermo Scientific) under the following settings: spray voltage, 1.6 kV, Capillary temperature, 275°C. Other settings should be empirically determined. **For unlabeled samples**: Raw data files should be searched against human protein sequences with the following parameters: Carbamidomethylation (+57.021 Da; cysteine) as static a modification. Oxidation/hydroxylation +15.995Da (methionine, proline, lysine), and deamidation +0.984 Da (asparagine, glutamine) as dynamic modifications. A minimum peptide length of five amino acids. A precursor mass tolerance of 15 ppm and fragment mass tolerance of 0.05 Da. Maximum false peptide discovery rate of 0.01. **For TMT-labeled samples**: The instrument should be operated in the data dependent mode to automatically switch between full scan MS and MS/MS acquisition. Survey full scan MS spectra (m/z 350−1800) should be acquired with 35,000 resolutions (m/z 200) after an accumulation of ions to a 3 × 10^6^ target value based on predictive automatic gain control (AGC). Maximum injection time should be 100 ms. The 20 most intense multiply charged ions (z ≥ 2) are sequentially isolated and fragmented in the octopole collision cell by higher-energy collisional dissociation (HCD) using normalized HCD collision energy 30 with an AGC target 1×10^5^ and a maxima injection time of 400 ms at 17,500 resolutions. The isolation window should be set to 2 and fixed first mass as 120 m/z. The dynamic exclusion should be set as 30 s. Charge state screening should be enabled to reject unassigned and 1+, 7+, and >7+ ions. Raw data files should be searched against human protein sequences with the following parameters: Carbamidomethylation (+57.021 Da; cysteine) and TMT labeling (+229.163 Da; N-terminus, lysine) set as static modifications. Oxidation/hydroxylation +15.995Da (methionine, proline, lysine), and deamidation +0.984 Da (asparagine, glutamine) set as dynamic modifications. A minimum peptide length of five amino acids. A precursor mass tolerance of 15 ppm and fragment mass tolerance of 0.05 Da. Maximum false peptide discovery rate of 0.01.

The following analysis assumes Proteome Discoverer (Thermo Fisher Scientific) is the proteomics tool utilized to analyze mass spectra, specifically utilizing the calculated protein peptide spectrum matches (PSMs) and/or abundance between control and treatment samples. Other analysis tools can be utilized, such as the freely available MaxQuant software. Analysis of unlabeled and TMT-labeling experiments is unique.

***Optional:*** Final protein candidates can be filtered against the Contaminant Repository for Affinity Purification (CRAPome).^9^ We utilized an arbitrary candidate exclusion threshold that removes any candidate that is detected in over 99 (out of 716) control experiments within the CRAPome database.

### Unlabeled experiments

Proximal interactor candidates are selected based on a series of calculations that compare PSMs and abundance between fusion protein samples and controls. Values for each protein across all samples should be organized into a spreadsheet. A template is provided with full calculations (Table S1; Tab 1).

### TMT-labeled experiments

Proximal interactor candidates are selected by initial normalization of calculated absorbance values for each protein (Table S1; Tab 2), followed by fold change comparison of samples versus the individual control. A fold change cut-off of 1.5 is applied, accounting for skew in favor of control samples through ratio compression^10^ and sample normalization prior to labeling and analysis. A template is provided with full calculations (Table S1; Tab 2).

## Limitations

Engineered biotin ligase-target fusion proteins may exhibit unnatural localization, structure, activity, and interacting partners. Biotin depletion/sequestration can induce cell stress, in particular when occurring over long periods. This protocol requires high levels of fusion protein and biotin ligase expression. Excess biotin ligase expression can induce toxicity^6^ and excess target expression may induce toxicity, growth arrest, enhanced proliferation, and/or differentiation. Proximal protein labeling via biotinylation requires available, exposed amino groups. If not using the ‘conditioned media transfer’ method, background labeling of intracellular targets is very high. In our studies, post-pulldown protein concentration was low (approximately 2.5 μg total protein). Sample handling thereafter should be treated as low input and handled with care. Use of trypsin-resistant streptavidin does not release biotinylated peptides following digestion, reducing overall coverage in peptide searches. Structural components of the extracellular matrix are often large, complexed, insoluble, and difficult to handle; these may be poorly represented in ePL studies.

## Troubleshooting

### Problem 1

Poor transgene expression/secretion (fusion gene cloning and cell line generation).

### Potential solution

Review gene transfer system protocol. Some cell lines may fare better with a lentiviral transfer method. Although transient transfection does work, cell recovery and expansion is not amenable to the extended assay time.

### Problem 2

No unique biotinylation detected through streptavidin blotting: biotin ligase versus biotin ligase-free samples (optimize proximity labeling conditions).

### Potential solution

All biotin ligase containing samples should exhibit off-target labeling that can be visualized through long exposure in streptavidin-HRP blots. Confirm that this is occurring versus biotin ligase-free samples and/or control samples without biotin. If no unique biotinylation is detected again, revisit early protocol steps assessing protein secretion via immunoblotting.

### Problem 3

No unique biotinylation detected through streptavidin blotting: fusion protein samples versus the biotin ligase control sample (optimize proximity labeling conditions).

### Potential solution

The signal to noise ratio may be low, making it difficult to observe unique patterns of biotinylation. It is possible that control and fusion protein expression is too high. Revisit early protocol steps assessing protein secretion via immunoblotting (optimize proximity labeling conditions).

### Problem 4

No biotinylation of a known interactor detected: fusion protein samples versus the biotin ligase control sample (optimize proximity labeling conditions).

### Potential solution

Endogenous known interactors may favor interaction with endogenous protein (for example, maybe the interaction initiates secretory vesicles). By including a recombinant known interactor, users can test the functionality of their ePL assay. The recombinant known interactor should be detected post-pulldown in samples (and not detected in control samples). If no unique biotinylation is detected again, revisit early protocol steps assessing protein secretion via immunoblotting.

### Problem 5

Poor peptide detection in TMT-labeled experiments (TMT-labeled processing).

### Potential solution

Make sure TMT labels are fresh, or were prepared and immediately frozen at −80°C within 30 days before use.

## Resource availability

### Lead contact

Further information requests should be directed to David Peeney (david.peeney@nih.gov).

### Technical contact

Further technical information requests should be directed to David Peeney (david.peeney@nih.gov).

### Materials availability

Plasmids and strains are available upon request.

### Data and code availability

The dataset generated during this study are available in the associated publication,^1^ and through MassIVE MSV000095637.

## Acknowledgments

This research was supported (in part) by the Intramural Research Program of the NIH, grant ZIA BC011204 (W.G.S.-S.).

Plasmids were constructed by the National Institute on Drug Abuse Genetic Engineering and Viral Vector Core Facility (RRID:SCR_022969).

## Author contributions

D.P. and W.G.S.-S. conceived the study. D.P. and S.G. developed the protocol. D.P., S.G., and J.A.R. performed the bulk of the experiments. S.C.-P., Y.L., and A.G. supported experimental goals. J.A.R. and D.P. wrote the manuscript. D.P. and W.G.S.-S. provided supervision. W.G.S.-S. acquired funding.

## Declaration of interests

The authors declare no competing interests.
